# Increased Lower Limb Loading During Sit-to-Stand is Important for the Potential for Walking Progression in Ambulatory Individuals with Spinal Cord Injury

**DOI:** 10.21315/mjms2019.26.1.9

**Published:** 2019-02-28

**Authors:** Lalita Khuna, Lugkana Mato, Pipatana Amatachaya, Thiwabhorn Thaweewannakij, Sugalya Amatachaya

**Affiliations:** 1 School of Physical Therapy, Faculty of Associated Medical Sciences, Khon Kaen University, Khon Kaen, Thailand; 2 Improvement of Physical Performance and Quality of Life (IPQ) Research Group, Khon Kaen University, Khon Kaen, Thailand; 3 School of Mechanical Engineering, Faculty of Engineering and Architecture, Rajamangala University of Technology Isan, Nakhon Ratchasima, Thailand

**Keywords:** rehabilitation, physiotherapy, walker, cane, walking device

## Abstract

**Background:**

Decreased rehabilitation time may increase the need for walking devices at the time of discharge to promote levels of independence among ambulatory individuals with spinal cord injury (SCI). However, using walking devices could create adverse effects on patients. This study explores the proportion of walking devices used, potential for walking progression, and associated factors among ambulatory individuals with SCI.

**Methods:**

Fifty-seven participants were assessed for their demographics and functional ability relating to the requirement for walking devices, including the Timed Up and Go Test (TUGT) and lower limb loading during sit-to-stand (LLL-STS).

**Results:**

Thirty-five participants (61%) used a walking device, particularly a standard walker, for daily walking. More than half of them (*n* = 23, 66%) had potential of walking progression (i.e., safely walk with a less-support device than the usual one). The ability of walking progression was significantly associated with a mild severity of injury, increased lower-limb muscle strength, decreased time to complete the TUGT, and, in particular, increased LLL-STS.

**Conclusion:**

A large proportion of ambulatory individuals with SCI have the potential for walking progression, which may increase their level of independence and minimise the appearance of disability. Strategies to promote LLL-STS are important for this progression.

## Introduction

Spinal cord injury (SCI) could introduce various degrees of functional limitation, psychosocial problems, medical complications, a burden of care on family, and loss of productivity (1, 2). The current dramatic decrease in rehabilitation length may further affect an optimal ability of the patients at the time of discharge. This subsequently increases the need for a walking device, particularly a standard walker, for ambulatory individuals with SCI to promote their level of independence and experience of task-specific walking practice and reduce the burden of care on their families (2). Later, the lack of a periodic follow-up tool may result in many patients still using the same walking device, even though their functional ability has improved. This could enhance the possible negative impact on the patients of longlasting use of a walking device, such as learning abnormal walking patterns (e.g., increased flexion posture and asymmetrical walking pattern); increased risk of musculoskeletal problems in the upper limbs and back; and elevated energy, attention, and neuromotor demands while walking (3–7). These impacts may further affect the appearance of disability and the ability of the patient to interact with their community, especially when the patients are using walking devices with high support ability (8, 9).

In contrast, walking with a less-supportive device when patients are not ready to do so may retard their walking speed and stability and increase energy expenditures that further distort the safety and community participation of patients (2). Thus, the exploration of the need for a walking device, the ability of walking progression, and factors associated with this progression in ambulatory individuals with SCI may suggest rehabilitation strategies to promote levels of independence and safety and minimise the appearance of disability for these individuals. Therefore, this study investigated the proportion of ambulatory individuals with SCI who needed a walking device, those who had potential for walking progression, and factors associated with this progression among these individuals.

## Materials and Methods

### Participants

This cross-sectional study was conducted among ambulatory participants with incomplete SCI in a tertiary rehabilitation centre and many communities in Thailand. The sample size was estimated from the number required for a major study (10), where p_1_ = 0.6 (6), the power of test = 90%, and the alpha = 0.05. The inclusion criteria were that the patient must be at least 18 years of age and have an SCI from traumatic causes or non-progressive diseases, with the ability to walk independently, with or without a walking device, continuously over at least 15 m (the Functional Independent Measure-Locomotion score = 5–7), and can follow the commands used in the study. Patients were excluded if they had any conditions that might affect ambulatory ability (e.g., brain function disorders; visual deficits; musculoskeletal pain with an intensity of more than 5 out of 10, according to a visual analogue scale; deformity of the spine or the lower extremities; or unstable medical conditions). Eligible participants signed a written informed-consent document approved by the local ethics committee [HE 571017], prior to taking part in this study.

### Protocols

Participants were interviewed and assessed for their demographics (including age, gender, weight, and height) and SCI characteristics (including post-injury time and cause, level and severity of SCI, using sensorimotor scores and the criteria from the American Spinal Cord Injury Association Impairment Scale or the AIS protocol) (11). Then, participants were interviewed for their level of fear of falling, using five rating scales: 1 = not at all, 2 = mild, 3 = moderate, 4 = very, 5 = most (12, 13).

Subsequently, the participants were assessed for their usual walking ability and types of walking devices required (if any). Then participants who used a walking device were assessed for the possibility of walking progression by gradually changing to a less-supportive walking device or no walking device, according to their optimal ability. Participants who could progress their walking ability safely—as determined by an experienced physiotherapist (i.e., more than 5 years of working experience in research and rehabilitation relating to patients with SCI) were categorised into the ‘able’ group; if not, they were classified into the ‘unable’ group (2). Then, participants were assessed for their ability relating to walking progression, including lower limb loading during sit-to-stand (LLL-STS) and the Timed Up and Go test (TUGT) using the following protocols:

#### Lower limb loading during sit-to-stand

The sit-to-stand (STS) is a very biomechanically demanding task for the lower limbs and balance ability; where a walking device is commonly used to reduce lower limb loading and promote balance ability (6, 10, 15). Hence, the LLL-STS might be an important factor relating to the progression of walking ability.

Participants were seated on a standard, armless chair with their back upright at 90° against the backrest of the chair, hip flexion at 90°, feet placed flat on a digital-load cell [Model L6E3-C, 200 kg-3G, maximum capacity 200 kg with accuracy of 35 g] (7, 10, 14, 15) at 10 cm behind the knees, and their arms at their sides or on parallel bars. Then, participants were instructed to stand up from the chair and attempt to take most of their body weight onto their legs, with or without using their arms. An average finding over three trials was used for data analysis.

#### Timed up and go test

TUGT is a valid and reliable functional mobility test that comprises many subtasks needed in daily living (intraclass correlation coefficient [ICC] = 0.997 to 1.000; *ρ* = −0.76 to −0.88). The outcomes can clearly discriminate ability of ambulatory individuals with SCI who did and did not fall (6, 12, 13, 16–18). The test recorded the time taken to rise from a standard armrest chair, walk along a 3 m walkway, and return to sit down on the chair, at a fast and safe speed with—or without—a customary walking device (17–19). An average finding over the three trials was used for data analysis.

During the tests, participants had a lightweight safety belt fastened around their waist and an assessor always alongside them to ensure their safety. Participants could take a period of rest, as needed, while participating in the study.

### Data Analysis

Descriptive statistics were used to explain the demographics, SCI characteristics, and findings of the study. The findings from all participants were used to represent the proportion of walking devices used. Then the data of those who used a walking device were categorised into two groups, including those who were “able” or “unable” to progress their walking ability. The Mann-Whitney U test and the Chi-square test were applied, to compare the findings between the groups, for continuous and categorical data, respectively. The multiple logistic regression analysis (odds ratio [OR], with corresponding 95% confidence intervals [95% CI]) was applied, to determine the factors associated with the potential for walking progression. The level of statistical significance was set at *P* < 0.05.

## Results

Sixty independent ambulatory participants with incomplete SCI were involved in the study. However, three participants did not complete the tests required for the study; thus, they were excluded and the data of 57 participants were reported. Most participants were male (*n* = 44, 77%), had a non-traumatic SCI (*n* = 33, 58%) with mild lesion severity (AIS D) (*n* = 41, 72%), and a chronic stage of injury (*n* = 50, 88%) (Table 1). Thirty-five participants (62%) normally used a walking device on daily basis, including a standard walker (30%), crutches (11%), or a single cane (21%) (Table 2).

Among those who used a walking device (*n* = 35), 23 participants (65%) had potential to progress their walking ability in which 14 participants could even walk without a walking device (Table 2). These participants (*n* = 23) had lower extremity muscle strength, LLL-STS, and time to complete the TUGT significantly better than those who were unable to progress their walking ability (*P* < 0.001), whereas the sensory scores and levels of fear of fall showed no significant differences between the groups (*P* > 0.05, Table 3). Factors associated with the potential for walking progression included having AIS D, increased lower extremity muscle strength and LLL-STS, and reduced time needed to complete the TUGT (*P* < 0.01, Table 4). Among these variables, the LLL-STS was the only significant factor in the final multivariate regression model (*P* < 0.05, Table 4).

## Discussion

This study assessed the proportion of walking devices used and those who were able to progress their walking ability—as well as factors associated with this progression—among ambulatory participants with SCI. The findings support the previous data (2, 6) that more than half of ambulatory individuals with SCI (62%) used a walking device for daily walking, and approximately two-thirds of them (65%) were capable of walking better than their usual ability.

Brotherton et al. (20) suggest that improved walking ability is associated with increased life satisfaction and physical function of the patients. However, Kim et al. (2) found that walking at the highest ability obviously reduced walking speed and increased energy expenditure, which could subsequently affect their patients’ safety and their ability to participate in their communities. Therefore, this study further explored factors associated with ability of walking progression, with the aim of suggesting crucial strategies for the safe and efficient walking progression of patients. The findings indicate that ability of walking progression was significantly associated with having AIS D, increased lower extremity muscle strength, decreased time needed to complete the TUGT, and increased LLL-STS (Table 4).

Having AIS D inferred that the participants had mild sensorimotor deterioration (i.e., at least half of the key muscles below the neurological level of the injury had a muscle grade 3 or higher), and thus they had high potential for walking (21). Haubert et al. (22) emphasised that the preservation of lower extremity functions makes walking a realistic mobility goal for ambulatory patients with SCI. Other studies also reported that muscles around the hip (23) and knee extensor strength (24) are associated with ambulatory function. Data of the present study further suggests that increased lower extremity muscle strength of one grade was significantly afforded potential for walking progression by 1.19 times (95% CI: 1.06 to 1.34; *P* < 0.01; Table 4).

Moreover, ability of walking progression was significantly associated with decreased time needed to complete the TUGT (Table 4). The TUGT comprises several mobility subtasks that require stability and the ability to transfer body weight, in order to complete the tasks within a short duration. Outcomes of the test could clearly identify ambulatory individuals with SCI, who walked with or without a walking device and who did or did not fall (12, 13, 16). This study applied the TUGT to indicate functional mobility and risk of fall, which are important contributors to the level of independence of the participants. Although other tests, such as the Berg Balance Scale, could be used to achieve the same objectives outcomes, those tests are unable to discriminate ambulatory individuals with or without falls (25). With better functional mobility, lower extremity muscle strength, and lower limb support ability, participants required less support from the upper extremities. Therefore, they were able to use less-supportive walking devices or progress their walking ability (Table 4).

Moreover, the increased LLL-STS inferred that the participants needed less contribution from the upper limbs to complete a sit-to-stand movement. Previous studies reported that the ability of independent sit-to-stand movement without hands requires complex interaction from many contributors, similar to those necessary for independent walking: lower extremity muscle strength, balance, functional mobility, and sensorimotor integration, to move the body centre of mass from the three-point, stable base of support of a sitting position to the less-stable, two-point base of support over the extended lower extremities of an upright standing position (10, 14). Therefore, of all univariate significant factors, increased LLL-STS was the only significant factor in the final multivariate regression model (adjusted OR [95% CI): 1.11 [1.01 to 1.22]; *P* < 0.05, Table 4).

A recent study found heterogeneity among ambulatory individuals with SCI who used the same types of walking devices and the possibility for their walking progression (6). Kim et al. (2) also reported that many individuals with SCI were capable of ambulation at multi-levels. The present findings suggest the important role of LLL-STS to further afford levels of independence and minimised appearance of disability for these individuals, in various clinical and community settings.

Nonetheless, this study contained a small sample size, and the findings were cross-sectionally gathered under a realistic area of a rehabilitation room. With the power of test greater than 90%, the findings may provide an important clue to promote safe and efficient walking progression for ambulatory individuals with SCI. However, walking ability in a real-life situation of the patients still needs individual consideration for many other factors, such as their environmental, socioeconomic, and psychological constraints. Thus, a training program for walking at a new ability as they could be progressed, both in a rehabilitation setting and in their environmental conditions, is necessary. In addition, a further intervention study among a larger number of participants could confirm the importance of LLL-STS in clinical practice for these individuals.

## Conclusion

With special efforts to promote rehabilitation outcomes (i.e., increased levels of independence and minimised appearance of disability) for patients with SCI, the current findings suggest that approximately two-thirds of ambulatory individuals with SCI had potential for walking progression. The incorporation of LLL-STS in rehabilitation practice plays a crucial role in this progression.

## Figures and Tables

**Table 1:** Demographics and spinal cord injury characteristics of the participants

Variable	All participants (*n* = 57)	Unable^1^ (*n* = 12)	Able^1^ (*n* = 23)
Age (years): mean ± SD (95% CI)	51.5 ± 15.0	52.3 ± 18.9	56.3 ± 14.0
Body mass index (kg m^−2^): mean ± SD (95% CI)	22.7 ± 3.9	21.8 ± 4.1	23.9 ± 4.1
Post-injury time (months) : mean ± SD (95% CI)	57.3 ± 62.3	51.9 ± 61.9	57.5 ± 63.0
Gender^2^: male, *n* (%)	44 (77)	8 (67)	17 (74)
Cause^2^: non-traumatic, *n* (%)	33 (58)	5 (42)	16 (70)
Level of injury^2^: incomplete paraplegia, *n* (%)	42 (74)	9 (75)	21 (91)
Stage of injury^2^: chronic, *n* (%)	50 (88)	12 (100)	20 (87)
AIS classification^2^: D, *n* (%)	41 (72)	2 (17)	18 (78)*

Notes:

1 Able = participants who were able to progress their walking ability to use a less support or without a walking device as compared to their usual ability Unable = participants who were unable to change their walking ability from their usual manner

2 These variables were categorised according to the following criteria: gender: male/female; cause: non-traumatic/traumatic; level of injury: incomplete paraplegia/incomplete tetraplegia; stage of injury: sub-acute (≤ 12 months)/chronic (> 12 months); AIS class: C/D

* Indicated statistically significant difference between the ‘able’ and ‘unable’ groups (*P* < 0.001; from the Chi-square test)

AIS: American Spinal Injury Association Impairment Scale

**Table 2:** Walking ability of the participants as determined using their usual, and highest and safe walking ability

Usual walking ability^1^	Highest walking ability^2^			

	Walker	Crutches	Cane	No device
Walker (*n* = 17)	9 (26)	**3 (8)**	**3 (8)**	**2 (6)**
Crutches (*n* = 6)		2 (6)	**3(8)**	**1 (3)**
Cane (*n* = 12)			1 (3)	**11 (32)**
No device (*n* = 22)				

Notes:

1 Usual walking ability was determined according to the type of walking device that the participants normally used for their daily walking

2 Highest and safe walking ability was determined according to the ability that the participants could safely walk with the least support device with no more than contact guarding assist

Bold letters indicated the number of participants who were able to progress their walking ability from their usual ability

**Table 3:** Sensorimotor scores, functional ability and level of fear of falling among participants who were able and unable to progress their walking ability

Variable^1^	Unable (*n* = 12)	Able (*n* = 23)	Mean difference (95% CI)
**Motor scores**^2^			
Upper extremities (total = 50)	47.7 ± 5.1 (44.4–50.9)	49.2 ± 2.61 (48.1–50.3)	1.5 (−1.1 to 4.2)
Lower extremities (total = 50)	21.8 ± 8.7 (16.2–27.3)	35.6 ± 8.5 (31.9–39.2)	13.8 (7.6 to 20.0)*
**Sensory scores**^3^			
Upper extremities (total = 76)	70.7 ± 10.4 (64.1–77.3)	72.6 ± 5.9 (70.0–75.2)	1.9 (−3.6 to 7.5)
Lower extremities (total = 36)	22.3 ± 5.0 (19.2–25.5)	23.8 ± 6.0 (21.2–26.4)	1.5 (−2.7 to 5.6)
LLL-STS (%)	59.8 ± 18.2 (48.2–71.3)	96.9 ± 11.42 (91.9–101.8)	37.1 (26.9 to 47.3)*
TUGT (second)	58.9 ± 18.3 (47.2–70.3)	27.3 ± 12.03 (22.1–32.5)	−31.6 (−42.1 to −21.1)*
Fear of fall	4.0 ± 1.2 (3.1–4.8)	3.4 ± 1.41 (2.8–4.0)	−0.6 (−1.5 to 0.4)

Notes:

1 All variables were reported using mean ± standard deviation (95% confidence interval)

2 Muscle strength of the key muscles from the methods of manual muscle testing of the 10 paired myotomes from the upper extremities (C5 to T1) and lower extremities (L2 to S1)

3 Tactile scores of the 28 dermatomes of upper extremities (from C2 to T12) and lower extremities (from L1 to S4–5) on the right and left sides of the body

* Indicated statistically significant differences between the groups (*P* < 0.001; from the Mann–Whitney U test)

LLL-STS: Lower-limb loading during sit-to-stand, TUGT: Timed Up and Go test

**Table 4:** Factors associated with the ability of walking progression of the participants

Variable	β (SE)	Unadjusted OR (95% CI)	*P*-value	β (SE)	Adjusted OR (95% CI)	*P*-value
Age (years)	0.02 (0.02)	1.02 (0.97–1.06)	0.479	−0.10 (0.19)	0.90 (0.62–1.31)	0.584
Body mass index (kg m^−2^)	0.14 (0.10)	1.15 (0.95–1.41)	0.158			
Post-injury time (months)	0.00 (0.01)	1.00 (0.99–1.01)	0.797			
AIS classification 1	2.89 (0.92)	18.00 (2.94–110.31)	0.002**	1.56 (3.45)	4.74 (0.01–4346.5)	0.655
Motor scores						
Upper extremities	0.11 (0.10)	1.12 (0.92–1.36)	0.261			
Lower extremities	0.18 (0.06)	1.19 (1.06–1.34)	0.003**	0.35 (0.31)	1.42 (0.77–2.61)	0.260
Sensory scores						
Upper extremities	0.03 (0.05)	1.03 (0.94–1.13)	0.476			
Lower extremities	0.05 (0.07)	1.05 (0.92–1.20)	0.467			
LLL-STS (%)	0.14 (0.05)	1.15 (1.05–1.26)	0.003**	0.10 (0.05)	1.11 (1.01–1.21)	0.029*
TUGT (second)	−0.14 (0.05)	0.87 (0.79–0.95)	0.004**	−0.09 (0.06)	0.92 (0.82–1.02)	0.112
Fear of fall	−0.35 (0.30)	0.70 (0.39–1.27)	0.244			

Notes:

1 AIS class C is a reference group

* Odds ratio is significantly different from 1.0; *P* < 0.05

** Odds ratio is significantly different from 1.0; *P* < 0.01

β: beta co-efficient, SE: standard error, OR: odds ratio, CI: confidence interval, AIS: American Spinal Injury Association Impairment Scale, LLL-STS: Lower-limb loading during sit-to-stand, TUGT: Timed Up and Go Test
